# Modeling Analysis on Germination and Seedling Growth Using Ultrasound Seed Pretreatment in Switchgrass

**DOI:** 10.1371/journal.pone.0047204

**Published:** 2012-10-10

**Authors:** Quanzhen Wang, Guo Chen, Hayixia Yersaiyiti, Yuan Liu, Jian Cui, Chunhui Wu, Yunwei Zhang, Xueqing He

**Affiliations:** 1 Department of Grassland Science, College of Animal Science and Technology, Northwest A&F University, Yangling, Shaanxi Province, People’s Republic of China; 2 Department of Plant Science, College of Life Science, Northwest A&F University, Yangling, Shaanxi Province, People’s Republic of China; 3 Institute of Grassland Science, College of Animal Science and Technology, China Agricultural University, Beijing, People’s Republic of China; 4 College of Pastoral Agriculture Science and Technology, Lanzhou University, Lanzhou, People’s Republic of China; Institut Mediterrani dEstudis Avançats (CSIC/UIB), Spain

## Abstract

Switchgrass is a perennial C4 plant with great potential as a bioenergy source and, thus, a high demand for establishment from seed. This research investigated the effects of ultrasound treatment on germination and seedling growth in switchgrass. Using an orthogonal matrix design, conditions for the ultrasound pretreatment in switchgrass seed, including sonication time (factor A), sonication temperature (factor B) and ultrasound output power (factor C), were optimized for germinating and stimulating seedling growth (indicated as plumular and radicular lengths) through modeling analysis. The results indicate that sonication temperature (B) was the most effective factor for germination, whereas output power (C) had the largest effect on seedling growth when ultrasound treatment was used. Combined with the analyses of range, variance and models, the final optimal ultrasonic treatment conditions were sonication for 22.5 min at 39.7°C and at an output power of 348 W, which provided the greatest germination percentage and best seedling growth. For this study, the orthogonal matrix design was an efficient method for optimizing the conditions of ultrasound seed treatment on switchgrass. The electrical conductivity of seed leachates in three experimental groups (control, soaked in water only, and ultrasound treatment) was determined to investigate the effects of ultrasound on seeds and eliminate the effect of water in the ultrasound treatments. The results showed that the electrical conductivity of seed leachates during either ultrasound treatment or water bath treatment was significantly higher than that of the control, and that the ultrasound treatment had positive effects on switchgrass seeds.

## Introduction

Recently, interest in biofuels has grown rapidly in response to the rising costs of fossil fuels and increasing public concern about environmental issues such as climate change. Switchgrass (*Panicum virgatum* L.), a productive warm-season pasture grass, is a perennial C4 plant that thrives in a wide range of North America habitats [1], [2] and has been identified as an important crop for biomass energy because of its abundant biomass [3], [4], excellent nutrient use efficiency, favorable feedstock costs [5], [6], and broad adaptability [7]–[10]. Switchgrass contains abundant sugars in the form of cellulose and hemicellulose, which can be converted to bioethanol by hydrolysis and subsequent fermentation [11], [12]. Therefore, switchgrass productivity affects its value for conversion to energy. Seed dormancy and slow seedling establishment are two major concerns in switchgrass production, often resulting in a poor stand with reduced productivity [13].

Many researchers have studied methods for improving germination and seedling growth, such as seed priming, hardening, humidification, growth regulators and dry heat treatments [14]–[16]. Many treatments also have been used for switchgrass. When soaked in 5.25% sodium hypochlorite for 15 min or 18 M H_2_SO_4_ for 10 min or 0.2% KNO_3_ for 14 days, switchgrass seed vigor improved [17], [18], and sodium nitroprusside significantly promoted seed germination at 25°C [19].

Seed priming is effective for partially hydrating the seed to a point where the germination processes begin but are not completed. Various seed priming techniques have been developed, including hydropriming (soaking in water), halopriming (soaking in inorganic salt solutions), osmopriming (soaking in solutions of different organic osmotica), thermopriming (treatment of seed with low or high temperatures), solid-matrix priming (treatment of seed with solid matrices), and biopriming (hydration using biological compounds) [14]. When switchgrass seeds were mixed with particulate solid matrix materials, treatment at 17°C for 2 days and wet-chill treatment at 4°C for 14 days, seedling emergence increased by 35% and 150%, respectively. Shen found that germination can be increased up to ≥80% with 14 d of stratification [20]. However, such seed priming was usually longer than 2 days, and some priming methods require nearly half a month, which is time-consuming. By contrast, ultrasound treatment, which requires only an ultrasound generator, can cause heat effects, mechanical effects, and chemical effects on seeds within a short time. Ultrasound treatments have been reported to stimulate germination in many different types of plants, such as *Calanthe* hybrids, bean [21], corn [22], barley [23], fern spores [24], alfalfa and broccoli [25], chickpea, wheat, watermelon and pepper [26], [27]. Methods to enhance switchgrass seed germination and seedling growth might be effective; however, there is no information available about the effects of ultrasound on switchgrass seed.

This research investigated the effects of ultrasound treatment on germination and seedling growth in switchgrass. To optimize the conditions for ultrasound treatment, four levels of three parameters (sonication time, sonication temperature, and ultrasound output power) were considered via orthogonal array. Orthogonal array design, as a chemometric method for optimizing an analytical procedure, has been adopted in various areas [28]. This method may minimize the assay number and time required. Moreover, considering any pair of columns, all possible combinations of factor levels appear and within the same times; orthogonal designs are balanced and separable, which provides reliable and optimal results [28]. Therefore, the orthogonal matrix design of L_16_(4^5^) was used in this research, and the germination percentage (GP), plumular length (PL) and radicular length (RL) were used as the experimental indicators [29].

## Results

### Orthogonal Analyses

The results of orthogonal experiments revealed that the highest PL and RL in the sixteen treatments were observed at conditions of A2B2C2 (sonication at 35°C, ultrasound output power of 300 W and a sonication time of 15 min) (Tables 1 and 2). The highest GP in the sixteen treatments was under conditions of A3B3C2 (sonication at 45°C, ultrasound output power of 300 W and a sonication time of 25 min) (Tables 1 and 2).

**Table 1:** Assignment of the levels and factors in the experimental design using an orthogonal matrix L_16_ (4^5^).

Factors*	A (min)	B (°C)	C (W)	D(vacancy)
Level I	5	25	200	–
Level II	15	35	300	–
Level III	25	45	400	–
Level IV	35	55	500	–

* Columns A, B and C represent the sonication time, sonication temperature and ultrasound output power, respectively. Column D stands for vacancy to account for the statistical error.

**Table 2:** The L_16_ (4^5^) matrix associated with the analytical results.

Treatments	A (min)	B (°C)	C (W)	D (vacancy)	Germinationpercentage (%)	Plumularlength (mm)	Radicularlength (mm)
1	1	1	4	3	66.92±15.44e	36.77±9.24def	11.39±4.21 h
2	2	1	1	1	75.31±11.10de	37.47±9.89cdef	13.34±5.03defg
3	3	1	3	4	77.62±19.60cd	38.53±8.93bcd	14.98±6.74bcd
4	4	1	2	2	81.08±14.38bcd	38.64±9.20bcd	14.14±6.63cde
5	1	2	3	2	83.08±16.44abcd	37.56±9.48cde	14.13±5.89cde
6	2	2	2	4	88.77±12.59ab	**41.33±9.57a**	**18.40±7.57a**
7	3	2	4	1	83.00±17.58abcd	39.55±9.42abc	16.28±6.71b
8	4	2	1	3	83.69±15.39abcd	38.56±9.25bcd	13.34±6.10defg
9	1	3	1	4	81.00±19.50bcd	35.60±9.02ef	12.35±5.02fgh
10	2	3	4	2	84.69±13.08abc	39.69±8.68abc	15.47±5.92bc
11	3	3	2	3	**90.12±11.44a**	41.05±9.32ab	**18.32±7.59a**
12	4	3	3	1	79.69±15.04cd	38.36±9.31cd	14.09±7.16cde
13	1	4	2	1	76.07±17.05cd	38.02±8.55cde	13.38±5.83defg
14	2	4	3	3	77.38±11.80cd	37.59±9.82cde	13.15±7.04efg
15	3	4	1	2	81.85±19.09abcd	36.84±10.02def	13.72±6.29def
16	4	4	4	4	77.23±24.97cd	34.91±8.52f	11.87±4.57gh

Values are the means ± S.D. of the experiments. Means with the same lower letters are not significantly different at P<0.05. The experiment was calculated for three replicates. The first experiment consisted of four subsamples (16×4 dishes in all); The second and third experiments consisted of three subsamples, respectively (16×3×2×2 dishes in all). The total samples of GP were 160. Each subsample of PL and RL was respectively calculated for ten seeds. PL and RL of 2560 seeds (16×4×10+16×3×2×2×10) were respectively measured. The highest values of GP, PL and RL are highlighted in bold for the 16 treatments.

The results of the range analyses showed that factor B (sonication temperature) had the highest R value (9.40) in GP (Table 3), whereas the range of factor A (sonication time) and C (ultrasound output power) were respectively highest in RL (Table 4) and PL (Table 5). We inferred that C (ultrasound output power) exhibited the largest effect on PL (Table 4) growth but B had the largest effect on GP (Table 3). By contrast, the effect of factor A (sonication time) on RL had the largest R, whereas GP and PL had the lower R values, respectively. The impact of the factors on GP, PL and RL (in decreasing order) were B- A- C, C- B- A, and A- C- B, respectively (Tables 3 to [Table pone-0047204-t004] 5).

**Table 3:** Average responses of each level and range of Germination percentage (%). N = 40.

Factor	A	B		C		D	
Level 1	76.77±18.03b		75.23±16.09b		80.46±16.65ab		78.52±15.49a
Level 2	81.54±13.17a		**84.63**±15.57a		**84.01**±14.97a		82.67±15.74a
Level 3	**83.14**±17.58a		83.88±15.37a		79.44±15.90b		79.53±15.96a
Level 4	80.42±17.87ab		78.13±18.68b		77.96±19.35b		81.15±19.91a
Range*	6.37		9.40		6.05		4.15
Order**	2		1		3		4

* The highest Germination percentage of the levels are highlighted in bold; Means with the same lower letters are not significantly difference at P<0.05.

** The ordinal numeral for the range sequence of the three factors in decreasing order.

**Table 4 pone-0047204-t004:** Average responses of each level and range of Radicular length (mm). N = 400.

Factor	A		B	C		D	
Level 1	12.81±5.36b	13.46±5.88b			13.19±5.64c		14.27±6.33a
Level 2	15.09±6.78a	**15.54**±6.86a			**16.06**±7.28a		14.37±6.20a
Level 3	**15.83**±7.02a	15.06±6.82a			14.09±6.73b		14.05±6.83a
Level 4	13.36±6.23b	13.03±6.02b			13.75±5.83bc		14.40±6.61a
Range*	3.02	2.51			2.87		0.35
Order**	1	3			2		4

* The highest Radicular length of the levels are highlighted in bold; Means with the same lower letters are not significantly difference at P<0.05.

** The ordinal numeral for the range sequence of the three factors in decreasing order.

**Table 5:** Average responses of each level and range of Plumular length (mm). N = 400.

Factor	A		B		C		D
Level 1	36.99±9.09b	37.85±9.32b		37.12±9.58c		38.35±9.30ab	
Level 2	**39.20**±9.60a	**39.25**±9.50a		**39.76**±9.25a		38.18±9.38ab	
Level 3	**38.99**±9.52a	38.68±9.27a		38.01±9.36b		38.49±9.51a	
Level 4	37.62±9.18b	36.84±9.30c		37.73±9.16bc		37.59±9.34b	
Range*	2.21	2.41		2.64		0.9	
Order**	3	2		1		4	

* The highest Plumular length of the levels are highlighted in bold; Means with the same lower letters are not significantly difference at P<0.05.

** The ordinal numeral for the range sequence of the three factors in decreasing order.

According to the orthogonal method, the highest level of the averages corresponded to the optimal conditions [28]. The calculated conditions of A3B3C2 (sonication time of 25 min, sonication temperature of 45°C, and output power of 300W) were optimal for GP and RL, whereas the conditions of A2B2C2 (sonication time of 15 min, sonication temperature of 35°C, and output power of 300W) was optimal for PL and RL (Table 2).

The variance analysis showed that factors A, B, and C were significant in GP, PL and RL (p<0.01), and there were significant coupling effects both pair-wise and among the three factors (Table 6). The multivariate analysis of variance was significantly different (Table 7). Therefore, binary quadratic regressions were performed to investigate the coupling effects of pair-wise factors and to assess for more accurate optimum conditions.

**Table 6:** Variance analyses for the model, for each experimental factors and among them.

Source (factors)	DF	Germination percentage (%)		Plumular length (mm)		Radicular length (mm)	
		F Value	Pr>F	F Value	Pr>F	F Value	Pr>F
A	3	23.22	<.0001	14.02	<.0001	34.74	<.0001
A*A	3	3.76	0.0110	6.43	0.0003	21.67	<.0001
B	3	65.31	<.0001	14.87	<.0001	25.39	<.0001
B*B	3	8.78	<.0001	6.72	0.0002	14.48	<.0001
C	3	20.99	<.0001	17.42	<.0001	26.89	<.0001
C*C	3	2.66	0.0229	4.83	0.0024	8.59	<.0001
D	3	2.43	**0.0649**	2.12	**0.0958**	0.49	**0.7335**
E	15	24.51	<.0001	10.22	<.0001	17.63	<.0001
F	2	1227.17	<.0001	1374.08	<.0001	282.06	<.0001
A*B	9	4.24	<.0001	7.40	<.0001	9.34	<.0001
B*C	9	5.21	<.0001	6.27	<.0001	11.96	<.0001
A*C	9	4.13	<.0001	6.55	<.0001	8.84	<.0001
Model	56	8.99	<.0001	27.65	<.0001	24.32	<.0001

* A, B and C represent the sonication time, sonication temperature and ultrasound output power, respectively.

**Table 7:** Multivariate Analysis of Variance - MANOVA Test Criteria and F Approximations for the Hypothesis of No Overall g Effect.

Statistic	Value	F Value	Num DF	Den DF	Pr > F
Wilks’ Lambda	0.35553379	535.25	4	3162	<.0001
Pillai’s Trace	0.67105241	399.42	4	3164	<.0001
Hotelling-LawleyTrace	1.73789388	686.76	4	1896.2	<.0001
Roy’s GreatestRoot	1.69374420	1339.75	2	1582	<.0001

H  =  Type III SSCP Matrix for g. E  =  Error SSCP Matrix. S = 2, M = −0.5, N = 789.5. F Statistic for Roy’s Greatest Root is an upper bound. F Statistic for Wilks’ Lambda is exact.

### Model Analysis

**Figure 1:**
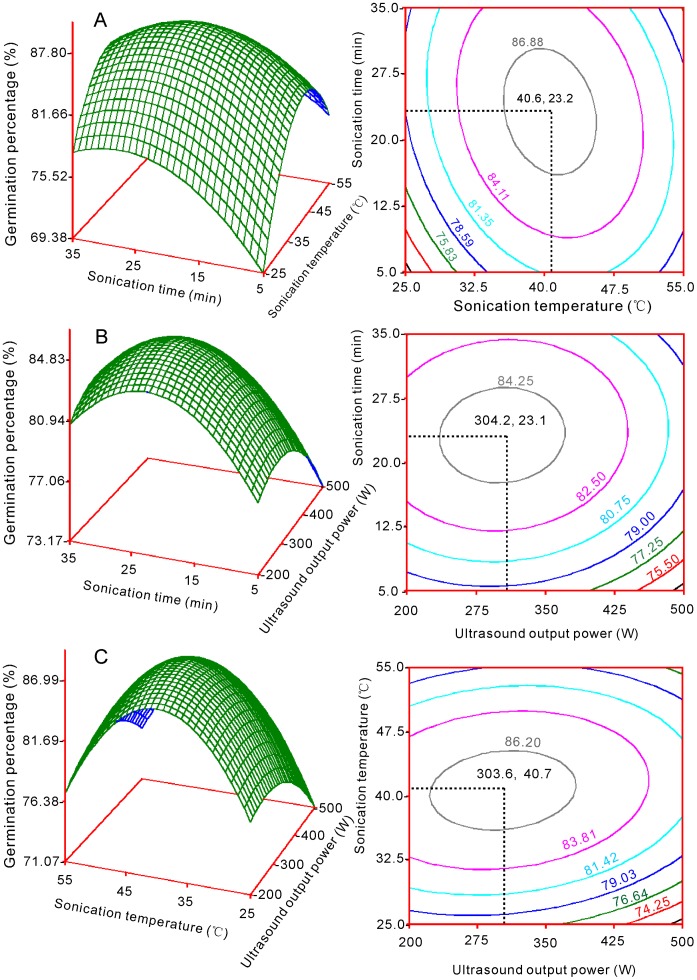
Response surface plots showing the parameter effects on the germination percentage in switchgrass by ultrasound irradiation. (a) Germination percentage vs. sonication time and sonication temperature; (b) Germination percentage vs. sonication time and ultrasound output power; (c) Germination percentage vs. sonication temperature and ultrasound output power.

**Figure 2:**
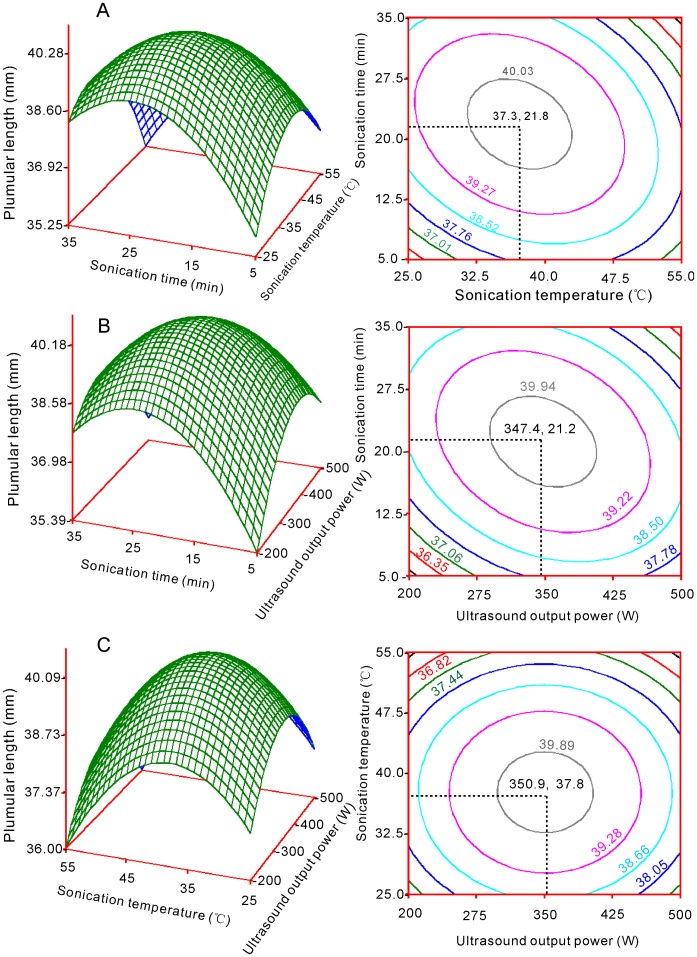
Response surface plots showing the parameter effects on the plumular length in switchgrass by ultrasound irradiation. (a) Plumular length vs. sonication time and sonication temperature; (b) Plumular length vs. sonication time and ultrasound output power; (c) Plumular length vs. sonication temperature and ultrasound output power.

**Figure 3:**
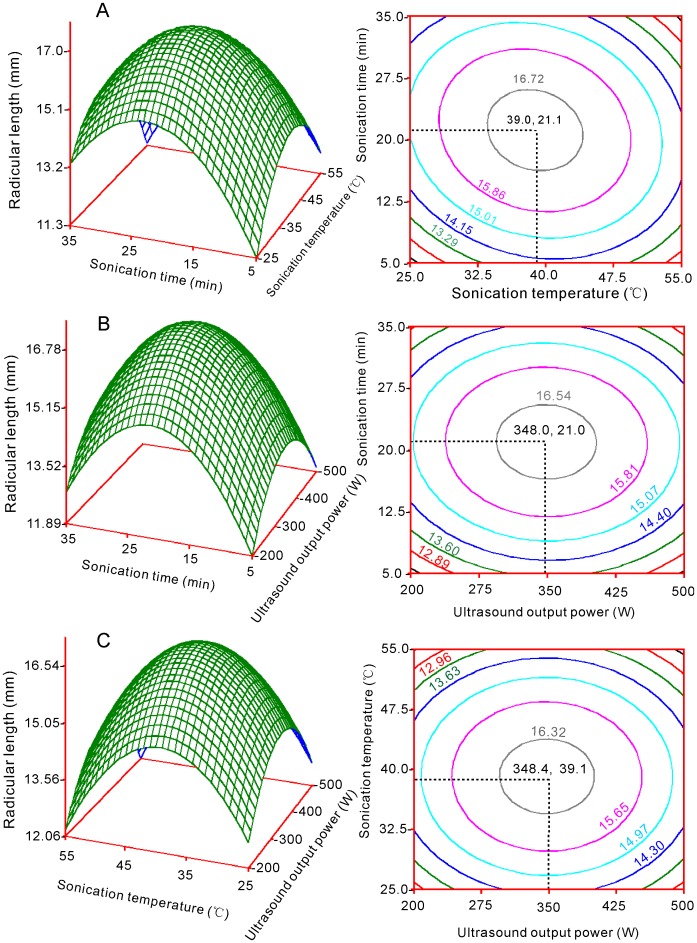
Response surface plots showing the parameter effects on radicular length in switchgrass by ultrasound irradiation. (a) Radicular length vs. sonication time and sonication temperature; (b) Radicular length vs. sonication time and ultrasound output power; (c) Radicular length vs. sonication temperature and ultrasound output power.

Quadratic polynomial models were used to accurately optimize conditions. Response surface graphs were respectively plotted with pair-wise variables for the experimental ranges (Figs. 1, 2 and 3). The response surfaces show the effects of sonication time with each of the two other factors on GP, PL and RL (Fig. 1 a, b, Fig. 2 a, b and Fig. 3 a, b). For factors A (sonication time) and B (sonication temperature), GP, PL and RL reached maxima at 23.2 min (Fig. 1a), 21.1 min (Fig. 2 a) and 21.8 min (Fig. 3 a), respectively. For factors A and C (ultrasound output power), GP, PL and RL reached maxima at 23.1 min (Fig. 1b), 21.0 min (Fig. 2 b) and 21.2 min (Fig. 3 b) respectively.

The effects of factor B and its interactions with the other two factors on GP, PL and RL are shown in Fig. 1a, c, Fig. 2a, c and Fig. 3a, c. The peak points for GP with factors B and A (Fig. 1a) or factors B and C (Fig. 1c) were 40.6°C and 40.7°C, respectively. The peak points for PL with factors B and A (Fig. 2a) or factors B and C (Fig. 2c) were 37.3°C and 37.8°C, respectively. The optimal sonication temperature peaks for RL were 39.0°C (Fig. 3a) and 39.1°C (Fig. 3c).

The effects of factor C are shown in Fig. 1b, c, Fig. 2 b, c and Fig. 3 b, c. The peak points for GP were 304.2 W and 303.6 W for factors C and A (Fig. 2b) and factors B and C, respectively, whereas the peak point for PL was 347.4 W if factors C and A were considered (Fig. 2b) and 350.9 W if factors B and C were considered (Fig. 2c). For RL, the optimal ultrasound output power peaks were 348.0 W and 348.4 W, when factors C and A (Fig. 2b) and B and C were respectively considered (Figs. 3 b and 3 c).

### Optimized Conditions

Six optimal values were obtained from the response surface analysis for each factor (Figs. 1, 2 and 3). The mode was the statistically thickest value. Therefore, the mode was used as the final optimal value. For factor A (sonication time), the six values were 23.2, 23.1, 21.8, 21.2, 21.1 and 21.0 min; the mode was 22.5. For factor B (sonication temperature), the six values were 40.6, 40.7, 37.3, 37.8, 39.0 and 39.1°C; the mode was 39.7. For factor C (ultrasound output power), the six values were 304.2, 303.6, 347.4, 350.9, 348.0 and 348.4 W. The mode was 348. Thus, the final optimal conditions were sonication for 22.5 min, sonication temperature at 39.7°C, and output power of 348 W.

### Test of the Sonication Effects (Experiment 2)

**Figure 4:**
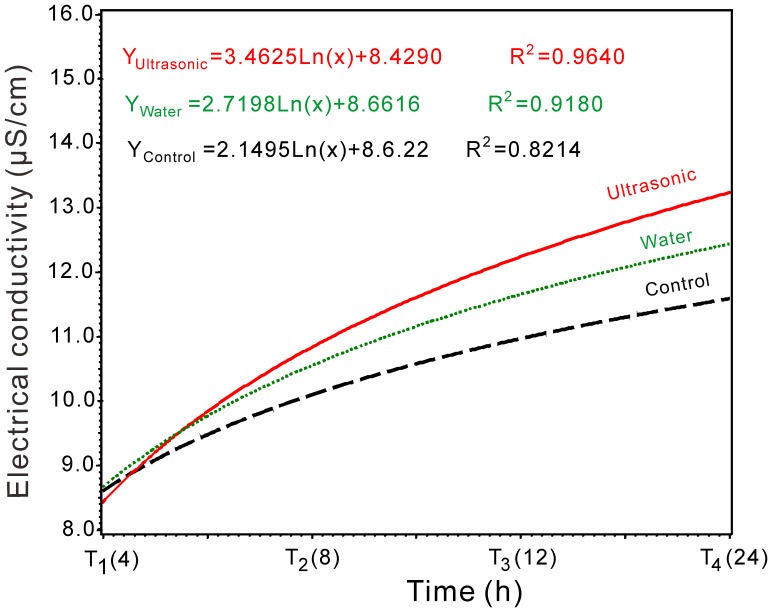
Electrical conductivity of switchgrass seeds soaked in water only (Water), soaked in water in ultrasonic device (Ultrasonic) and without any treatment (Control).

To investigate the effects of ultrasound on seed while eliminating the effect of water in the ultrasound treatments, the electrical conductivity of seed leachates in the three experimental groups (CONTROL, water, and ultrasonic) were determined (Table 8). The optimal conditions were used in the ultrasound treatment, and temperature and time of the water treatment were the same as those of the ultrasound treatment. The algorithmic models generated from the experimental data of electrical conductivity of seed leachates were estimated using the logarithmic models shown in Fig. 4. The regression models of electrical conductivity of seed leachates agreed with the experimental results, with R-squared values of 0.821, 0.918, and 0.964, respectively.

**Table 8:** Electrical conductivity of switchgrass^e^ of the three treatment groups: control, seeds soaked in water and soaked in water in ultrasonic device after 4 to 24 h of soaking.

Time after soaking	4 (h)	8 (h)	12 (h)	24 (h)
Control	8.31^b^	10.40^c^	11.63^c^	10.90^c^
Soaks in water only	8.48^a^	10.64^b^	12.30^b^	11.87^b^
Ultrasound treatment^f^	8.33^b^	10.80^a^	12.78^a^	12.81^a^

e Values are the means of three repetitions in the experiments. Means within the columns (indicated by different letters) are significantly different at P<0.05.

f Water bath was used when seeds were exposed to ultrasound.

The results in Table 8 show that with all the treatments, electrical conductivity of seed leachates significantly (p<0.05) increased with time. The electrical conductivity of the water bath treatment was significantly greater than the control after 4, 8, 12, 24 h of soaking. The electrical conductivity of the ultrasound treatment was significantly greater than the water bath treatment after 8, 12, 24 h of soaking (p<0.05), meaning that both water and ultrasound have effects on switchgrass seed during the ultrasound treatment.

## Discussion

Many different mechanisms have been suggested to explain the effect of ultrasound on seeds. The main conclusion is that when ultrasound is used, pressure fluctuations cause the formation, growth, and violent collapse of microbubbles in the sonication liquid. The bubble collapse leads to physical, biological and chemical changes in seed. Ultrasound treatment increased the moisture content of chickpea, wheat, pepper and watermelon seeds early during treatment periods [27]. Such results suggest that enhancement of seed germination is the result of mechanical or shear effects from large and rapid oscillations in bubble size, which lead to the disruption of plant cell walls and thereby increase water uptake by the cells. Additionally, alpha-amylase levels of barley seeds with ultrasound treatment were greater than that of the control [30]. Scouten reported that in combination with chemicals and heat, ultrasound treatment of alfalfa seeds at 38.5 to 40.5 kHz could improve the killing of *Salmonella* and *E. coli* O157:H7, which live in alfalfa seeds [31].

Because the seeds were soaked in water, it is possible that both water and ultrasound were important during the ultrasound treatment. The effect of water on seeds has been reported [32]–[34]. Soaking seeds in water before sowing and drying after soaking, which is called hydropriming, is a simple method to enhance seed germination and seedling emergence. Hydropriming reduces the inhibitory activities of trypsin-like proteolytic enzymes, increases á-amylase activity, and alters the mobilization of both inorganic and organic substances from storage organs to the developing embryo in some species [14]. These results coincide with the results of this study that ultrasound treatments improve the germination and enhance the seedling growth of switchgrass.

During a slow hydration process, membranes reorganize to attain their original structure, which increases membrane integrity. Rapid hydration may cause leakage of essential nutrients from, damaging the seed and allowing the leaching of sugars, amino acids, and minerals [35]. Electrical conductivity of seed leachates is usually negatively related to seed vigor [36]. Electrical conductivity of both ultrasound and water bath treatments were significantly greater than those of the control after 8, 12 and 24 h of soaking (p<0.05) in this study, but the plumular length of switchgrass seedling in sixteen treatments were longer than that of the control (25.75 mm). It is possible that ultrasonic treatments have both positive effects and negative effects on switchgrass seeds, and the negative effects may be caused by rapid hydration; however, the positive effects had a stronger impact than the negative effects in this research.

There is a wide range of appropriate conditions for ultrasound treatment in different plants: 60W, 22°C, and 2 min for spruce [37]; 460W, 30°C, and 15 min for barley [38]; 135W and less than 7 min for *Calanthe* hybrids [26]; and 45 min for chickpeas [27]. This range may be due to seed characters (such as thickness of seed coat, size, and dormancy), which vary depending on the species. In this study, the final optimal conditions were sonication for 24 min at 41°C and at an output power of 320 W.

All of the surface plots were convex, and maxima were apparent in the central region (Figures 1, 2 and 3). Furthermore, most peak values were close to those obtained from the range analysis, which indicates that the optimal conditions by model analysis are reliable. Based on these results, each optimal level of factors was not high and was set in the middle, which suggested that dual effects may exist during ultrasound treatment. When a longer time, greater temperature, or ultrasound output power is used, physical or chemical damage occurs, which is caused by the pressure induced by the ultrasonic wave. Ultrasound treatments longer than 5 min had a negative impact on pepper germination rates [27]. The heavily damaged region was observed within 200 min from the ginger particle surface when ultrasound was used [39]. Moreover, one study reported that prolonged treatment time (≥7 min) increased the percentage of destroyed embryos of seeds, which leads to decreased germination rates [26].

### Conclusions

In this study, an orthogonal matrix design was an efficient method for optimizing the conditions of ultrasound treatment on switchgrass seeds. Sonication temperature (B) was the most effective factor for germination, whereas the output power (C) exhibited the largest effect on seedling growth for the ultrasound treatment. Combined with the analysis of range, variance and models, the optimal ultrasonic treatment conditions for improving germination and seedling growth were sonication for 22.5 min at 39.7°C and at an output power of 348W. The electrical conductivity of seed leachates from either ultrasound treatment or water bath treatment were significantly greater than those of the control at 8, 12 and 24 h of soaking (p<0.05), which demonstrated that ultrasound has positive effects on switchgrass seeds, improving seedling growth. In conclusion, the results above provide a basic evidence for applying ultrasound to pretreat switchgrass seeds. In addition, as a simple, cheap and time saving method, ultrasound treatment has the potential to be used in improving seedling growth, thereby improving the final yield of switchgrass.

## Materials and Methods

Two experiments were performed. One experiment evaluated the sonication effect on switchgrass germination and seedling growth using an orthogonal matrix design. The other experiment investigated the mechanism of the effects on seeds. In the second trial, the electrical conductivity of seed leachates under optimal treatment, water-soaking treatment, and control treatment were investigated. Untreated seeds were used as a control. Because the seeds were soaked in water when ultrasound was used, the water-soaking treatment, whose temperature and time were the same as those for ultrasonic treatment, was also performed to eliminate the effect of water in the ultrasound treatments.

### Seed Materials and Instrumentation

Switchgrass seed materials were gotten from The Institute of Soil and Water Conservation of Chinese Academy and China Agricultural University. This study was performed at the Laboratory of Grassland Science Department, Northwest A&F University Shaanxi Province, China). The varieties of the plant were Alamo and Summer.

Ultrasonic irradiation was produced by an ultrasound generator (KQ-500DE, Kunshan Ultrasound Instrument Co., Ltd., China). The ultrasound generator had a fixed frequency of 40 kHz, an adjustable temperature ranging from 10 to 80°C, a sonication bath capacity of 22.5 L (500 × 300 × 150 mm) with water inlet and outlet valves, and an adjustable ultrasound output power from 200 to 500 W. In addition, an electro-thermal constant-temperature oven (DHG-9140A, Shanghai Yiheng Instrument Co., Ltd., China), a plant incubator (ZPW-400, Harbin DongTou SG-Tech Development Co., Ltd., China) and an electronic analytical balance (YP1200, Shanghai Science and Industrial Co., Ltd., China) were used.

### Experimental Design

Three ultrasonic factors were selected, including sonication time (factor A), sonication temperature (factor B) and ultrasound output power (factor C). According to the orthogonal matrix design [L_16_ (4^5^)], each of the three ultrasonic irradiation factors was assigned four levels (Table 1), and sixteen treatment combinations at different parameters were established [28] (Table 2).

### Seed Treatments

According to the experimental design for the conditions described in Table 2, the samples (100 seeds for each treatment) coated by gauze were immersed in distilled water in the ultrasound generator. The water temperature was heated to the temperature level required before immersion. The ultrasound treatments were performed at 40 kHz in the ultrasonic generator with the corresponding conditions. The temperature of the circulating water was set and checked intermittently, such that it remained constant during the experiments.

### Germination Tests

Three times of germination experiments with 16 treatments were respectively conducted. Each of them was run immediately after sixteen different treatments with germination at 25°C in 100 mm Petri dishes (100 seeds per dish) on two layers of filter paper moistened with 5 ml of distilled water. Germination percentage and plumular and radicular lengths were measured on day 7. Using the variety Alama, the first experiment consisted of four subsamples of each treatment (16×4 dishes all together). Using both variety Summer and Sunrise, the second and third experiments consisted of three subsamples, respectively (16×3×2×2 dishes all together). 10 germinated seeds were randomly selected from each Petri dish for measuring the lengths of plumules and radicles. Therefore, 16 dishes (4+3×2×2) of each treatment were evaluated for germination percentage; 160 seedlings of each treatment were evaluated for plumular and radicular lengthes.

### Tests of the Sonicated Effects (Experiment 2)

To investigate the mechanisms of the sonication effects and eliminate the effect of water in the ultrasound treatments, the electrical conductivity of seed leachates in the three experimental groups (i.e., control, water, and ultrasonic) were determined. The optimized conditions elucidated from the orthogonal array design were used in the ultrasound treatments. To eliminate the effect of water in the ultrasound treatments, parallel treatments were performed in which seeds were soaked in water only. The tests without any treatment were the control experiments (Control). The seeds for these experiments (Control, water, and ultrasonic) were surface-dried and adjusted to their original moisture content at room temperature, as determined by the changes in seed weight.

To determine the electrical conductivity of seed leachates, fifty seeds from the three experimental groups were soaked in disposable plastic cups containing 20 mL of deionized water for 4, 8, 12 and 24 hours at 25°C (Table 8). Readings were made using a conductivity meter.

### Statistical Analysis

Data were subjected to analysis of variance (ANOVA) using the SAS software (version 8.2) [40]. Differences between the means were tested by the Student-Newman-Keuls test, and values of p<0.05 were considered significantly different.

For generic results, the variables (factors A, B and C) were denoted by X_1_, X_2_ and X_3_. The dependent variables, the GP, PL and RL were denoted by Y_1_, Y_2_ and Y_3_, respectively. These variables were approached and analyzed via pair-wise, variable (X_1_ and X_2_, X_1_ and X_3_, and X_2_ and X_3_) quadratic regression models [41]–[43]:
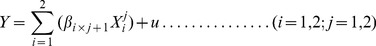
(1)where β is constant.

Response surface and contour charts are respectively graphed for Y_1_, Y_2_ and Y_3_ with their corresponding variables (X_1_, X_2_ and X_3_) (Figs. 1, 2 and 3). The analyses and graphical procedures specified above were performed using SAS (v8.2) [30].

Six responding values were respectively obtained from the quadratic analysis for each factor (A, B and C). The mode was considered as the thickest value [42]. The mode of the six values was calculated using the following formulas:
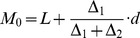
(2)

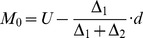
(3)where L is the lower-limit value of the array where the mode was located. U is the upper limit value of the array where the mode was located. △_1_ is the distance of the frequency between the lower adjacent array and the mode array. △_2_ is a distance of the frequency between the upper adjacent array and the mode array; *d* is the distance between the arrays.
